# Editorial: Impact of physical activity on health and behavioral risks in adolescents

**DOI:** 10.3389/fspor.2026.1828473

**Published:** 2026-04-10

**Authors:** Yijie Peng, Xin Xu, Yibo Wu

**Affiliations:** 1 Department of Nursing, The Fourth Affiliated Hospital of School of Medicine, and International School of Medicine, International Institutes of Medicine, Zhejiang University, Yiwu, China; 2 Guanghua School of Management, Peking University, Beijing, China; 3 School of Nursing, Henan University of Science and Technology, Luoyang, China

**Keywords:** physical activity, adolescents, health risk behaviors, mental well-being, physical fitness

## 1 Introduction

Over 80% of adolescents worldwide do not meet recommended physical activity guidelines [1]. This coincides with rising sedentary behavior, excessive screen time, and worsening mental health indicators among youth [2–5]. Physical activity patterns formed during adolescence tend to persist into adulthood [6, 7], making the relationship between physical activity and health risk behaviors in this age group worth close examination.

This Research Topic compiles 21 contributions examining how physical activity relates to behavioral risks, mental well-being, and physical fitness. Although the Topic is framed around adolescent health, the included studies span a broader developmental range from younger adolescents to college-age young adults, reflecting the continuity of health behavior formation across the transition to emerging adulthood. We organize the contributions around two themes: (1) how physical activity relates to risk behaviors, psychological well-being, and fitness, (2) the family, school, and social contexts that shape engagement.

Table 1 summarizes the key characteristics and findings of the 21 contributions.

**Table 1:** Summary of contributions in this research topic.

No.	Authors	Study design/participants	Key findings
Physical Activity, Risk Behaviors, and Well-Being			
1	Jia et al.	Network meta-analysis (24 RCTs, young adults, China)	Mind-body exercises (e.g., yoga, tai chi) reduce internet addiction symptoms
2	Said & Alibrahim	Cross-sectional (N=2,815 adolescents, Saudi Arabia)	28.4% overweight/obese; sedentary behavior identified as key risk correlate
3	Stratakis et al.	Cross-sectional (N=1,266 medical students, Serbia)	Dose-response relationship between PA and anxiety reduction
4	Wang et al.	Cross-sectional (N=1,075 college students, China)	Rumination and mindfulness mediate PA–subjective wellbeing pathway
5	Li et al.	Cross-sectional (N=1,308 college students, China)	Exercise attitudes and sleep quality chain-mediate PA–happiness link
6	Ma et al.	Cross-sectional (N=9,504 adolescents, China)	Anxiety mediates PA–quality of life link; gender moderates the pathway
7	Yan et al.	Systematic review and meta-analysis (8 RCTs, adolescents)	Exercise interventions improve adolescent sleep quality
8	Chen et al.	RCT (N=92 college students, China)	Jogging and rope skipping have differential fitness effects
9	Li et al.	RCT (N=40 male university students, China)	8-week functional training improves movement and fitness vs. traditional training
10	Liu et al.	Controlled trial (N=52 adolescent sprinters aged 15–18, China)	Combined functional and traditional training optimizes sprint performance
11	Li et al.	Cross-sectional (N=14,735 university students, China)	Non-linear association between BMI and physical fitness
12	Su et al.	Instrument development (N=526 middle school students, China)	Novel 31-item Adolescent Health Behavior Checklist validated
Ecological Contexts: Family, School, and Social Support			
13	Wu et al.	Cross-sectional with IV (N=7,482 adolescents aged 12–18, China)	Adolescent PA associated with sleep quality, mediated by family sports behavior
14	Yu	Cross-sectional (*N* = 4,717 adolescents and their parents, China)	Maternal health literacy predicts adolescent PA more than paternal
15	Wang et al.	Meta-analysis of RCTs (10 studies, children under 13)	Family-centered interventions increase children’s MVPA
16	Tiwari et al.	Longitudinal (N=3.4M, grades 6–12, USA)	Improved school climate linked to higher PA levels across grade levels
17	Aly et al.	Multi-center intervention study (*N* = 937, ages 8–14, 5 Mediterranean countries)	School-based PA program improves fitness; greater gains in ages 11–14
18	Wu et al.	Cross-sectional (N=378 junior high students, China)	Online PE engagement promotes perceived physiological and psychological health
19	Zhai et al.	Computational modeling (secondary EEG datasets)	EEG-based model for performance measurement in PE
20	Niu et al.	Cross-sectional (N=489 college students, China)	Self-efficacy mediates social support–PA participation link
21	Qian et al.	Cross-sectional (N=6,913 adolescents, China)	Ethnic and residential disparities in 24-h movement guideline adherence

PA, physical activity; MVPA, moderate-to-vigorous physical activity; IV, instrumental variable; PE, physical education; EEG, electroencephalography; RCT, randomized controlled trial.

## 2 Physical activity, risk behaviors, and well-being

The contributions in this section examine how physical activity relates to youth health across three interconnected dimensions: behavioral risks such as internet addiction and sedentary lifestyles, psychological well-being including anxiety and life satisfaction, and physiological outcomes, particularly sleep quality and physical fitness, that underpin overall well-being.

At the behavioral level, Jia et al.’s network meta-analysis finds that mind-body exercises are associated with reduced internet addiction symptoms in young adults, suggesting that structured physical activity may serve as a constructive alternative to internet addiction. Extending this concern to sedentary lifestyles more broadly, Said and Alibrahim survey 2,815 Saudi adolescents and report that 28.4% were overweight or obese, identifying sedentary behavior as a key correlate.

These behavioral risks are closely tied to psychological well-being. Stratakis et al. identify a dose-response association between physical activity and anxiety reduction among medical students, suggesting that even modest increases in activity may yield mental health benefits. Several studies further unpack the mechanisms underlying this association. Wang et al. report that rumination and mindfulness mediate the link between physical activity and subjective well-being. Building on the role of cognitive appraisal, Li et al. find that exercise attitudes and sleep quality function as chain mediators between physical activity and happiness. Ma et al. propose a moderated mediation model in which anxiety mediates the link between physical activity and quality of life, with gender moderating this pathway. Together, these mediation studies suggest that physical activity influences psychological outcomes through multiple cognitive and affective pathways.

Physical activity also shapes physiological dimensions of well-being. Yan et al.’s meta-analysis confirms that exercise interventions lasting 3 to 12 weeks improve adolescent sleep quality, with the greatest benefits observed at 12 weeks. This finding reinforces the mediating role of sleep identified by Li et al. above. In the domain of physical fitness, different training modalities yield distinct benefits. Chen et al. compare jogging and rope skipping among college students and find differential fitness effects. Li et al. report improvements from an 8-week functional training program. Liu et al. evaluate functional, traditional, combined, and control conditions for adolescent sprinters, finding that the combined approach optimizes sprint performance. Complementing these intervention studies, Li et al. document associations between BMI and health-related fitness among university students, suggesting that the findings can inform public health strategies aimed at reducing obesity and enhancing physical fitness. Finally, Su et al. contribute a new Adolescent Health Behavior Checklist covering six dimensions—exercise, diet, personal responsibility, sleep, interpersonal relationships, and stress management—providing a comprehensive measurement tool for adolescent health behaviors.

## 3 Ecological contexts: family, school, and social support

Several contributions examine how family, school, and peer environments shape physical activity engagement. Wu et al. find that adolescent physical activity is associated with improved sleep quality, a relationship mediated by family sports behavior. Yu reports that maternal health literacy has a stronger link to adolescent physical activity than paternal literacy. Wang et al.’s meta-analysis supports the effectiveness of family-centered interventions for increasing physical activity in children under 13.

At the school level, Tiwari et al.’s longitudinal analysis shows that improved perceived school climate is linked to higher physical activity levels in middle and high school students. Aly et al. provide cross-national data from the DELICIOUS project across Mediterranean settings. Wu et al. find that online physical education can promote health behaviors when in-person instruction is unavailable. Zhai et al. propose an adaptive model integrating electroencephalography (EEG) analysis to capture neural markers of emotional and cognitive states during physical education, enabling more precise performance evaluation. Niu et al. show that social support is associated with greater physical activity participation, with self-efficacy as a mediator.

Not all adolescents have equal access to these ecological resources. Qian et al. document ethnic and residential differences in 24-h movement guideline adherence between Zhuang and Han adolescents in southern China, finding that while ethnic differences in the guideline–fitness association are minimal, notable disparities exist between urban, suburban, and rural settings.

## 4 Conclusion and future directions

Taken together, these 21 studies demonstrate that the relationship between physical activity and health in youth populations is being studied from multiple angles. The collection’s strengths include its methodological diversity, with evidence drawn from meta-analyses, randomized trials, longitudinal tracking, and cross-sectional surveys across several countries. The ecological perspective is well represented: studies address not only individual-level associations but also how families, schools, and peers shape physical activity engagement, and how access varies across ethnic and residential groups. The range of outcomes examined, from internet addiction and anxiety to sleep quality and physical fitness, reflects the breadth of pathways through which physical activity may matter for youth health.

At the same time, several limitations should be noted. First, most studies rely on cross-sectional designs, so the mediation models in this collection describe statistical patterns rather than confirmed causal sequences. Second, physical activity tends to co-occur with other health-promoting behaviors and may partly serve as a marker of broader lifestyle patterns or social resources, yet few studies here attempt to isolate its independent contribution. Third, although the Topic is framed around adolescents, a substantial proportion of studies draw on university or medical student samples. This likely reflects the practical ease of recruiting college-based populations, but it also highlights a gap: evidence remains thinner for middle and high school populations, where family and school constraints may shape physical activity differently. Finally, the coverage of behavioral risks is uneven, as internet addiction and sedentary behavior receive attention, but substance use, tobacco and alcohol consumption, and self-harm are largely absent from the collection.

Future work would benefit from longitudinal and experimental designs, broader sampling across younger age groups and geographic settings, and longer follow-up periods. Digital delivery of physical education, increasingly relevant in hybrid learning environments, also deserves closer study. We thank all authors and reviewers for their contributions to this Research Topic.
